# Crime and Perceived Neighborhood Safety Across Racial-Ethnic Groups

**DOI:** 10.1093/geroni/igab046.1319

**Published:** 2021-12-17

**Authors:** Alfredo Velasquez, Fangqi Guo, Jennifer Robinette

**Affiliations:** 1 Chapman University, Orange, California, United States; 2 Chapman University, Irvine, California, United States

## Abstract

Crime often increases safety concerns for residents, and safety concerns are generally associated with worse health. Despite that marginalized racial/ethnic groups are more likely than non-Hispanic Whites to live in areas with more crime, prior studies have documented that these groups differentially view crime as a threat to safety. Furthermore, older adults are more likely to report safety concerns than younger adults, despite a lesser chance of being victimized. Using multiple waves of data from the Health and Retirement Study, a representative sample of US adults aged 51 years and older (n= 11,161, mean age of 66 years), we conducted weighted repeated cross-sectional linear regressions to examine whether the association between crime and perceived neighborhood safety varies by racial/ethnic group, by age, or by wave of data collection. Study results indicated that higher crime rates consistently predicted more safety concerns among non-Hispanic Whites, Hispanics, and “Others,” but were inconsistently associated with safety concerns among non-Hispanic Blacks, adjusting for age, household wealth, and census tract-level concentrated disadvantage, population density, and racial/ethnic heterogeneity. Furthermore, among non-Hispanic Whites, greater crime predicted more safety concerns before, but not after including a measure of racial/ethnic heterogeneity. These patterns persisted across the full age span. Racial/ethnic differences in the crime-safety link could be explained by additional sociopolitical and environmental variables including diversity that vary over time. Follow-up analysis is needed to determine if the racial/ethnic differences in crime-safety links extend to health.

